# Cervical Pessary for Prevention of Preterm Birth: A Meta-Analysis

**DOI:** 10.1038/srep42560

**Published:** 2017-02-17

**Authors:** Xin-Hang Jin, Dan Li, Li-Li Huang

**Affiliations:** 1 Women’s Hospital, School of Medicine, Zhejiang University, Hangzhou, Zhejiang, China; 2 Wenzhou People’s Hospital, Wenzhou, Zhejiang, China

## Abstract

To investigate the efficacy of cervical pessary placement in preventing preterm birth and perinatal morbidity and mortality in asymptomatic women with a singleton pregnancy and a short cervix, we searched literature in relevant databases. The meta-analysis of the 3 included trials (1412 women) showed cervical pessary placement did not reduce the risk of spontaneous preterm birth <34 weeks in these women (risk ratio (RR), 0.71; 95% confidence interval (CI), 0.21–2.43, P = 0.59; I^2^ = 90%). The sensitivity analyses by excluding one trial at one time showed the same results. This meta-analysis also showed that cervical pessary did not prevent preterm birth <34, 30, 28 weeks and was not associated with respiratory distress syndrome, necrotising enterocolitis, intraventricular haemorrhage, neonatal sepsis, retinopathy of prematurity, fetal death, neonatal death, perinatal death, birth weight <1500 g, birth weight <2500 g, premature preterm rupture of membranes, corticosteroid treatment for fetal maturation, and admission to neonatal intensive care unit. Although this meta-analysis showed cervical pessary placement did not reduce the risk of preterm birth in women with a singleton pregnancy and a short cervix, we could not confirm or refute this conclusion, and large-scale randomised controlled trials are urgently needed.

Preterm birth is defined as any birth before 37 completed weeks of gestation, or fewer than 259 days of gestation^1^. Preterm birth is a major determinant of neonatal morbidity and mortality and has lifelong adverse consequences on health^2^. It is estimated that preterm birth accounts for more than one third of the 3.072 million neonatal deaths in the world in one year^3^. Preterm birth is also the leading cause of child death in nearly all middle and high-income countries^3^. Moreover, the morbidity associated with preterm birth such as cerebral palsy, learning disability, and chronic disease in adulthood results in enormous economic and social cost^1^,^4^.

Spontaneous preterm birth accounts for two thirds of preterm birth, and is also the most common cause of extremely preterm birth, which is any birth before 28 weeks, whereas the rest is medically indicated due to maternal or fetal complications such as preeclampsia, or intrauterine growth restriction^5^. Spontaneous preterm birth is a syndrome caused by multiple pathological processes such as inflammation, vascular disease, and disruption of maternal-fetal tolerance^6^. However, the precise cause of spontaneous preterm birth is unexplained in more than 50% of all the cases^1^. A short cervix, defined as a cervical length ≤25 mm on transvaginal ultrasound in the mid-trimester of pregnancy, which is one of the most important risk factor for preterm birth, has emerged as one of the strongest predictors of preterm birth in asymptomatic women with a singleton pregnancy^7^,^8^,^9^. Despite great efforts in research and treatment over the last few decades, preterm birth remains a formidable challenge to obstetricians.

Cervical pessary made of silicone or plastic, which is available in different shapes and sizes, has been used to prevent preterm birth in the past 50 years^10^. The precise mechanism of action by which cervical pessary may prevent preterm birth is largely unknown. One potential mechanism is that it functions in a mechanical manner by bending the cervix posteriorly. Thus, the pressure on the internal cervical ostium would be directed on the anterior lower uterine segment^11^. Another suggested mechanism is that by encompassing the cervix and compressing the cervical canal, the cervical pessary might protect cervical mucus plug, which plays an important role in pregnancy maintenance^11^.

A previous systematic review showed a need for more randomised trials to confirm the beneficial effects of cervical pessary in reducing preterm birth^12^. However, the latest trial indicated that cervical pessary did not result in a lower rate of spontaneous preterm birth in women with a singleton pregnancy and a short cervix compared with expectant treatment^13^. Therefore, we found it necessary to conduct a meta-analysis to evaluate the efficacy of cervical pessary placement in preventing preterm birth in women with a singleton pregnancy and a short cervix.

## Methods

### Search strategy

We conducted a systematic search for the relevant published literature without language restrictions until November 1st 2016 using the following databases: PubMed, the Cochrane Central Register of Controlled Trials, and Embase. The following medical subject heading (MeSH) terms, keywords, and their combinations were used: pessary; premature birth(‘preterm’, ‘premature’); premature labor(‘preterm’, ‘premature’). Appropriate suffixes were used for each database. We also manually searched the reference lists of the initially identified articles, previously published meta-analyses and reviews for additional relevant publications.

### Study selection and data extraction

We included randomised controlled trials comparing pessary therapy with expectant treatment for prevention of preterm birth in asymptomatic women with a singleton pregnancy and a short cervical length ≤25 mm as determined by ultrasonography in the mid-trimester. We excluded trials assessing cervical pessary placement in women with premature rupture of membranes, regular uterine contractions, or mid-trimester bleeding. Two independent reviewers screened the titles and abstracts to identify potentially eligible trials and then retrieved and assessed the full texts of the relevant citations for inclusion. The data extracted included the first author, year of publication, population characteristics, intervention details, reported outcomes, and study design. Data extraction was performed independently by two of the reviewers. Any disagreement between the two reviewers was solved unanimously through discussion. The Cochrane risk of bias tool has been used for risk assessment in included studies^14^. This tool assesses seven domains of risk of bias (random sequence generation, allocation concealment, blinding of participants and personnel, blinding of outcome assessment, incomplete outcome data, selective reporting and other bias), and categorises randomised trials by ‘low, unclear or high risk of bias’ in each domain.

### Outcomes

The primary outcome was spontaneous preterm birth <34 weeks of gestation and the secondary outcomes included preterm birth <34, <30 and <28 weeks of gestation; respiratory distress syndrome; necrotising enterocolitis; intraventricular haemorrhage; neonatal sepsis; retinopathy of prematurity; fetal death; neonatal death; perinatal death; birth weight <1500 g and <2500 g; premature preterm rupture of membranes; corticosteroid treatment for fetal maturation; and admission to neonatal intensive care unit.

### Data Synthesis

We reported the dichotomous data results after pooling estimates across trials with a random effects meta-analysis as RR with 95% CIs. Heterogeneity was assessed with the I^2^ statistic. We performed sensitivity analyses to evaluate the effect of risk of bias on the overall results by excluding one trial at one time. All analyses were done using Revman statistical software version 5.

## Results

**Figure 1:**
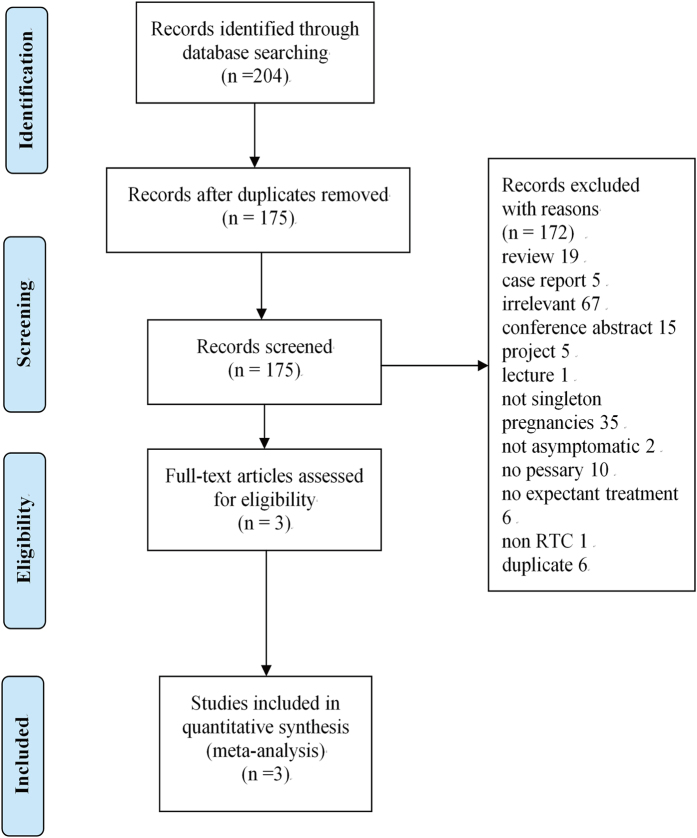
Study selection flow diagram.

Of the 204 citations identified, 29 were duplicates, and 172 were excluded based on title and abstract. After assessing full texts of the remaining 3 citations, we included 3 randomised controlled trials evaluating efficacy and safety of cervical pessary placement for prevention of preterm birth in this meta-analysis^13^,^15^,^16^. The PRISMA flow diagram illustrating the selection procedure is shown in Fig. 1.

Baseline characteristics of the 3 included trials is shown in Table 1.

**Figure 2:**
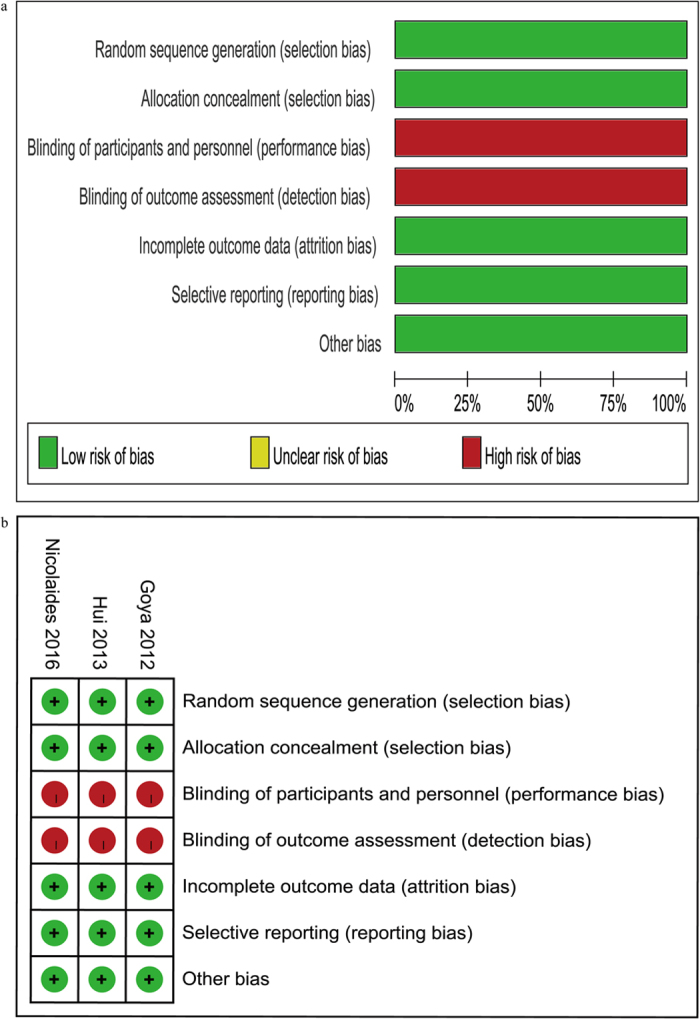
(**a**) Risk of bias graph, (**b**) Risk of bias summary (‘+’ low risk; ‘?’ unclear risk; ‘−’ high risk).

All the three trials were designed to evaluate the effect of cervical pessary placement on preterm birth in women with a singleton pregnancy and a short cervix^13^,^15^,^16^. Women with a sonographic cervical length <25 mm or ≤25 mm at 18–24 or 20–24 weeks of gestation were included. Women with abnormal conditions such as cervical cerclage, major fetal abnormalities were excluded. Maternal age in the three trials was similar. The women in one trial were Chinese^16^, and the women in the other two trials were mainly white^13^,^15^. All trials used the same type of cervical pessary. Cervical pessary placement was started at 18–22 weeks of gestation in one trial^15^, at 20–24 weeks of gestation in two trials^13^,^16^. Cervical pessary was removed at 37 weeks of gestation, or earlier under certain circumstances such as active vaginal bleeding, painful uterine contractions, and rupture of membranes. Although the primary outcome of the trial by Hui *et al*.^16^ differed from that of the other two trials^13^,^15^, they were actually the same, as all of the preterm births <34 weeks of gestation were spontaneous preterm births <34 weeks of gestation in this trial^16^. Control interventions were expectant treatment in these trials^13^,^15^,^16^. Assessment of risk of bias is shown in Fig. 2. Five domains (random sequence generation, allocation concealment, incomplete outcome data, selective reporting and other bias) were assessed as ‘low risk of bias’ in all trials, whereas two domains (blinding of participants and personnel, blinding of outcome assessment) were assessed as ‘high risk of bias’ due to the open-label nature of these trials.

### Primary outcome

#### Spontaneous preterm birth <34 weeks of gestation

**Figure 3:**
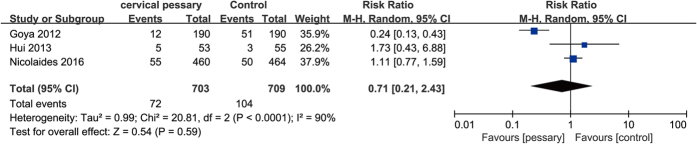
Efficacy of cervical pessary placement in preventing spontaneous preterm birth <34 weeks in women with a singleton pregnancy and a short cervix compared with control.

The meta-analysis of the 3 trials (1412 women) showed cervical pessary placement had no significant effect on spontaneous preterm birth <34 weeks of gestation in women with a singleton pregnancy and a short cervical length ≤25 mm compared with expectant treatment (RR 0.71; 95%CI, 0.21–2.43, P = 0.59; I^2^ = 90%) (Fig. 3).

Due to the high heterogeneity, we performed sensitivity analyses to explore the cause of heterogeneity. When we performed the sensitivity analyses by excluding one trial at one time, the results remained the same. However, when the trial by Goya *et al*.^15^ was excluded, the high heterogeneity was eliminated (RR 1.14, 95% CI 0.81–1.62, P = 0.46; I^2^ = 0%).

### Secondary outcome

The meta-analysis showed no statistically significant difference between the cervical pessary placement and control groups in preterm birth <34 weeks of gestation (RR 0.75; 95% CI 0.23–2.39, P = 0.62; I^2^ = 90%), <30 weeks of gestation (RR 1.24; 95% CI 0.78–1.96, P = 0.36; I^2^ = 0%), <28 weeks of gestation (RR 1.52; 95% CI 0.84–2.74, P = 0.16; I^2^ = 0%), respiratory distress syndrome (RR 0.79; 95% CI 0.22–2.90, P = 0.72; I^2^ = 82%), necrotising enterocolitis (RR 0.95; 95% CI 0.11–8.13, P = 0.96; I^2^ = 47%), intraventricular haemorrhage (RR 1.04; 95% CI 0.63–1.74, P = 0.87; I^2^ = 0%), neonatal sepsis (RR 0.67; 95% CI 0.23–1.96, P = 0.46; I^2^ = 69%), retinopathy of prematurity (RR 1.21; 95% CI 0.05–28.10, P = 0.91; I^2^ = 66%), fetal death (RR 1.61; 95% CI 0.53–4.90, P = 0.40), neonatal death (RR 1.32; 95% CI 0.48–3.66, P = 0.59; I^2^ = 0%), perinatal death (RR 1.33; 95% CI 0.65–2.76, P = 0.44; I^2^ = 0%), birth weight <1500 g (RR 0.72; 95% CI 0.18–2.84, P = 0.64; I^2^ = 90%), birth weight <2500 g (RR 0.60; 95% CI 0.16–2.17, P = 0.43; I^2^ = 95%), premature preterm rupture of membranes (RR 0.39; 95% CI 0.09–1.71, P = 0.21; I^2^ = 72%), corticosteroid treatment for fetal maturation (RR 0.75; 95% CI 0.47–1.18, P = 0.22; I^2^ = 37%), and admission to neonatal intensive care unit (RR 1.23; 95% CI 0.88–1.71, P = 0.23; I^2^ = 0%) (Table 2).

## Discussion

As far as we know, this is the first meta-analysis which combined all the available randomised trials to evaluate the efficacy of cervical pessary in preventing preterm birth and perinatal morbidity and mortality in asymptomatic women with a singleton pregnancy and a short cervix. However, this meta-analysis showed that cervical pessary did not reduce the risk of spontaneous preterm birth <34 weeks of gestation in these women compared with expectant treatment. The sensitivity analyses by excluding one trial at one time showed the same results. This meta-analysis also showed that cervical pessary did not prevent preterm birth <34, 30, 28 weeks of gestation and had no significant adverse effects on perinatal morbidity and mortality.

Cervical pessary is a device which is easily placed and removed without anesthesia, and is inexpensive compared with progesterone and cerclage. Unfortunately, this meta-analysis of the 3 trials showed cervical pessary had no beneficial efficacy in preventing preterm birth in women with a singleton pregnancy and a short cervix, but the heterogeneity is high. Therefore we analysed these trials in detail. First, we noticed that the preterm birth rates in the pessary group were similar in the three trials^13^,^15^,^16^, and the preterm birth rates in the control group were similar in two trials^13^,^16^. However, the preterm birth rate in the control group was much higher in the trial by Goya *et al*.^15^. In addition, according to the worldwide survey of preterm birth rates in 2010, the preterm birth rate in Spain was less than 10%^1^, and if we applied this data in this meta-analysis, the result would be the same but the high heterogeneity would be significantly reduced. Therefore, the results of the trial by Goya *et al*.^15^ could be a chance finding. Second, in the trial by Nicolaides *et al*.^13^, progesterone was used in 45% participants, and as progesterone has proven to be able to prevent preterm birth^17^,^18^,^19^,^20^, this might compromise the efficacy of cervical pessary. Hence, future large-scale multiple-center randomised trials are urgently needed, before cervical pessary can be generalised in clinical practice.

This meta-analysis showed that cervical pessary had no significant adverse effects on respiratory distress syndrome, necrotising enterocolitis, intraventricular haemorrhage, neonatal sepsis, and perinatal death, among others. No serious side effects were reported in these trials^13^,^15^,^16^, which is consistent with all the published studies^21^. The most common side effects of cervical pessary placement are an increase in vaginal discharge and pain during the pessary insertion and removal^21^. Moreover, in the trial by Goya *et al*.^15^, 95% of women in the pessary group recommended cervical pessary to others.

This meta-analysis has its limitations. The open-label nature of the trials could result in risk of bias for blinding of participants and personnel and outcome assessment, which hampers the validity of research of cervical pessary. Because of the inconsistence and scarcity of trials, we were unable to explore the effects of maternal age, ethnicity, obstetric history, body mass index, and cervical length on the results. In addition, none of the trials had the power to assess the treatment effect of cervical pessary.

To confirm the efficacy of cervical pessary in preventing preterm birth, future researches may focus on the following respects. First, as progesterone has been suggested to be effective in preventing preterm birth^17^,^18^,^19^,^20^, combination therapy such as cervical pessary and progesterone compared with progesterone may be considered. Second, although a short cervix is a high-risk factor in preterm birth, the cause of preterm birth is multiple factorial, basic research exploring the mechanisms behind preterm birth is still needed.

In conclusion, although this meta-analysis showed cervical pessary did not reduce the risk of preterm birth in women with a singleton pregnancy and a short cervix, we could not confirm or refute this conclusion, and large-scale randomised controlled trials are urgently needed.

## Additional Information

**How to cite this article:** Jin, X.-H. *et al*. Cervical Pessary for Prevention of Preterm Birth: A Meta-Analysis. *Sci. Rep.* **7**, 42560; doi: 10.1038/srep42560 (2017).

**Publisher's note:** Springer Nature remains neutral with regard to jurisdictional claims in published maps and institutional affiliations.

## Figures and Tables

**Table 1:** Characteristics of included trials. Interquartile range (IQR).

Trial(year)	Region	Inclusion criteria	Exclusion criteria	Age(pessary vs control)	Ethnicity	Intervention	Timing of treatment	Duration of treatment	Control	Primary outcome
Goya^15^	Spain	Women with singleton pregnancies and with a sonographic cervical length ≤25 mm	Major fetal abnormalities,painful regular uterine contractions,active vaginal bleeding,ruptured membranes, placenta previa, and a history of cone biopsy or cervical cerclage *in situ*	30.3 ± 5.1 vs 29.6 ± 5.4	White(56–58%) Latin American(29–31%) Other(13%)	Cervical pessary placement	18–22wk	Until 37 wk unless active vaginal bleeding, persistent contractions, or severe discomfort	Expectant management	Spontaneous preterm birth <34wk
Hui^16^	China	Women with singleton pregnancies and with a sonographic cervical length <25 mm	A history of cervical incompetence,surgical cerclage,major fetal abnormalities,cervical dilation,painful uterine contractions,rupture membranes	31.6 ± 4.7 vs 31.8 ± 5.3	Chinese	Cervical pessary placement	20–24wk	Until 37 wk unless active vaginal bleeding, painful contractions, or rupture of membranes	Expectant management	Preterm birth <34wk, the number of which equals that of spontaneous preterm birth <34wk
Nicolaides^13^	England, Slovenia, Portugal, Chile, Australia, Italy, Albania, Germany, and Belgium	Women with singleton pregnancies and with a sonographic cervical length ≤25 mm	Maternal age <16 years,fetal death,major fetal defect,cervical cerclage *in situ*,painful uterine contractions,ruptured membranes	Median(30.1 vs 29.5) IQR(26.0–34.2 vs 25.4–34.1)	White(63.9–67.9%) Black(26.3–28.8%) Asian(4.2–4.7%) Mixed(1.5–2.6%)	Cervical pessary placement	20–24wk	Until 37 wk unless medical termination of pregnancy,active vaginal bleeding,rupture of membranes,patient’s request,or preterm labor	Expectant management	Spontaneous preterm birth <34wk

**Table 2:** Efficacy of cervical pessary placement in preventing perinatal morbidity and mortality in women with a singleton pregnancy and a short cervix compared with control.

Secondary outcome	Trials	Pessary(Events/Total)	Control(Events/Total)	RR(95% CI)	P value	I^2^
preterm birth <34 wk	Goya^15^, Hui^16^, Nicolaides^13^	79/703	109/709	0.75 (0.23 to 2.39)	0.62	90%
preterm birth <30 wk	Hui^16^, Nicolaides^13^	38/513	31/519	1.24 (0.78 to 1.96)	0.36	0%
preterm birth <28 wk	Hui^16^, Nicolaides^13^	27/513	18/519	1.52 (0.84 to 2.74)	0.16	0%
respiratory distress syndrome	Goya^15^, Hui^16^, Nicolaides^13^	38/703	50/709	0.79 (0.22 to 2.90)	0.72	82%
necrotizing enterocolitis	Goya^15^, Nicolaides^13^	6/650	5/654	0.95 (0.11 to 8.13)	0.96	47%
intraventricular hemorrhage	Goya^15^, Hui^16^, Nicolaides^13^	28/703	28/709	1.04 (0.63 to 1.74)	0.87	0%
neonatal sepsis	Goya^15^, Hui^16^, Nicolaides^13^	33/703	37/709	0.67 (0.23 to 1.96)	0.46	69%
retinopathy of prematurity	Goya^15^, Nicolaides^13^	5/650	3/654	1.21 (0.05 to 28.10)	0.91	66%
fetal death	Goya^15^, Hui^16^, Nicolaides^13^	8/703	5/709	1.61 (0.53 to 4.90)	0.4	NA
neonatal death	Goya^15^, Hui^16^, Nicolaides^13^	8/703	6/709	1.32 (0.48 to 3.66)	0.59	0%
perinatal death	Goya^15^, Hui^16^, Nicolaides^13^	16/703	12/709	1.33 (0.65 to 2.76)	0.44	0%
birth weight <1500 g	Goya^15^, Nicolaides^13^	48/650	54/654	0.72 (0.18 to 2.84)	0.64	90%
birth weight <2500 g	Goya^15^, Nicolaides^13^	113/650	142/654	0.60 (0.16 to 2.17)	0.43	95%
premature preterm rupture of membranes	Goya^15^, Hui^16^	9/243	25/245	0.39 (0.09 to 1.71)	0.21	72%
corticosteroid treatment for fetal maturation	Goya^15^, Hui^16^	89/243	129/245	0.75 (0.47 to 1.18)	0.22	37%
admission to neonatal intensive care unit	Hui^16^, Nicolaides^13^	61/513	51/519	1.23 (0.88 to 1.71)	0.23	0%
